# Supporting second language acquisition of bilingual preschool children through professionalization of caregivers in specialized preschool programs

**DOI:** 10.3389/fpsyg.2023.1149447

**Published:** 2023-06-23

**Authors:** Jannika Boese, Julian Busch, Birgit Leyendecker, Anna-Lena Scherger

**Affiliations:** ^1^Research Unit of Language and Communication, Department of Rehabilitation Sciences, TU Dortmund University, Dortmund, Germany; ^2^Child and Family Research, Faculty of Psychology, Ruhr-University Bochum, Bochum, Germany

**Keywords:** professionalization, language support, early education, dialogic reading, bilingual children, specialized preschool programs

## Abstract

The aim of the present study was to investigate the effectiveness of a training program on language support strategies and dialogic reading for caregivers working in specialized preschool programs. These programs serve children without a regular childcare place who grow up with one or more languages other than German as the environmental language. Recent studies investigating the development of children attending these programs found only moderate improvements in German receptive language skills, while language support quality of the programs was rated as average. We assessed receptive second language competencies in vocabulary and grammar of *n* = 48 children and language support competencies of *n* = 15 caregivers using an interventional pre-posttest design. Receptive vocabulary skills of children supported by trained caregivers (intervention group) were compared to children supported by untrained caregivers (control group, *n* = 43). We found that both children’s and caregivers’ competencies increased from pre- to posttest, whereas the control group’s receptive vocabulary skills did not increase noticeably. The caregivers’ language support competencies influenced the increase of children’s receptive grammar but not vocabulary skills. The comparison between the intervention group and control group consistently showed no effect of group membership on children’s receptive vocabulary acquisition over time. Since the control group data came from a secondary analysis, only receptive vocabulary skills could be compared. The preliminary results of our study suggest that a caregivers’ training on language support strategies and dialogic reading in everyday educational situations support bilingual children’s grammar acquisition.

## 1. Introduction

Worldwide, there is an increasing number of children growing up bilingually. In Germany, 40% of all children under the age of six have a migration background (Autorengruppe Bildungsberichterstattung, 2022). Half of these children grow up with one or more languages other than German (Autorengruppe Bildungsberichterstattung, 2022). Bilingual children who receive little or no input in their second language [L2, German] during preschool years face the challenge of acquiring these language skills parallel to the academic skills required in schools when entering the new and unfamiliar school environment (Michalak et al., 2015). Therefore, children with a migration background are more often faced with an educational disadvantage compared to children without migration background (Tienda and Haskins, 2011; Forrell and Bellenberg, 2022). Although it has been demonstrated that low linguistic performance in the environmental language is associated with educational disadvantages (Ballantyne et al., 2008), it has been shown that (a) bilingualism has no negative influence on cognitive development (e.g., Bialystok et al., 2012; Wimmer and Scherger, 2022) and (b) low language achievements and disadvantages in the educational system seem more likely linked to low socioeconomic status [SES] (Pace et al., 2017; Voltmer et al., 2021; Stitzinger, 2022). In Germany, for instance, children with a migration background are three times more likely to grow up in a family at risk of social disadvantage and six times more likely to experience educational disadvantages compared to children without a migration background (Autorengruppe Bildungsberichterstattung, 2022).

Moreover, in Germany, almost one fifth of all children with a migration background does not enroll into daycare before entering school (Statistisches Bundesamt, 2021) due to a lack of childcare places. This is particularly problematic since longer daycare attendance is linked to better language outcomes of bilingual children in the environmental language (Ballantyne et al., 2008; Becker, 2010; Giesen et al., 2013). In order to promote social participation for bilingual children, it is important to provide them with high-quality L2 input before they enter school. Therefore, there are additional specialized early childhood development [ECD] programs for preschool children outside of regular daycare to compensate for the shortage of childcare places. These programs aim at a familiarization with basic cultural, school-relevant techniques prior to school start, above all the promotion of language skills. It has been shown that the quality of early childhood education [ECE] is decisive for how much children benefit from attending preschool (NICHD Early Child Care Research Network and Duncan, 2003).

In the present study, we implemented language support into these specialized ECD groups through video-based training of the caregivers working in these programs. Our aim was to investigate the effectiveness of a video-based training focusing on so-called language support strategies [LSS] used in daily routines such as dialogic reading [DR] and other highly structured everyday situations. As knowledge and skills are considered prerequisites for the performance of language support, we assessed language support knowledge and skills of participating caregivers using a standardized questionnaire. Furthermore, we assessed language outcomes of the promoted children within an interventional pre-posttest control group design.

### 1.1. Language Support and L2 Acquisition of Recently arrived immigrant children attending specialized ECD programs

Recent evidence on the links between language support and L2 acquisition among bilingual children with low length of exposure [LoE] to German comes from an investigation of specialized ECD groups. In response to the increased influx of immigrant and refugee populations to Germany in 2016, the federal government has started subsidizing specialized ECD programs for recently arrived immigrant children who could not enroll into other forms of daycare (Busch and Leyendecker, 2019). In a series of investigations, Busch and colleagues examined the implementation and quality of language support in the specialized ECD programs (Busch et al., 2023). The language support was better than in regular ECE groups according to a standardized rating scheme administered by licensed observers (Classroom Assessment Scoring System, Pre-Kindergarten; La Paro et al., 2002; Pianta et al., 2008), but still within the lower average range. Surprisingly, caregivers in the specialized programs had heterogenous ECE professionalization with more than 20 percent not reporting any ECE-related qualification. Compared to regular daycare, caregiver-child ratio in the specialized programs was better (1 caregiver for 3–4 children per group), frequency of children’s program attendance was lower (e.g., 4–5 days weekly with 3 h per day) and the caregivers were, on average, highly involved.

In a subsequent study, Busch and colleagues investigated the links between children’s German language acquisition and children’s attendance of the specialized ECD programs (Busch et al., 2021). The authors overall obtained inconsistent results. As expected, the recently arrived immigrant children had German language skills on very low levels. Longer periods of program attendance were linked to better German language skills and the authors observed within-child language improvements throughout a 5-month period of attendance. However, those children attending the specialized ECD groups did not show advantages over a control group of recently arrived immigrant children without experiences of formalized ECE. The work by Busch and colleagues thus provides preliminary evidence that caregivers with varying ECE professionalization might not apply effective language support to bilingual children. Still, their work has some decisive limitations. The methodological approaches were correlative and children of the control group design were slightly older than the group of children attending the specialized ECD groups. Further research is warranted to clarify the impact of caregivers’ daycare-embedded language support competencies and knowledge on the L2 acquisition among recently arrived immigrant children. Such research should especially ensure (a) staff’s professionalization regarding the improvement of language support competencies to foster language acquisition (i.e., LSS) through intensive training programs, (b) employ a research design that directly links staff’s knowledge and competencies on language support with child language acquisition, and (c) use control groups to verify the results.

### 1.2. Language support strategies and dialogic reading

LSS have their origin in early parent–child communication. Parents and caregivers intuitively apply certain techniques in their child-directed speech that are intended to support the child’s language acquisition. For example, in the first 2 years of life, parents support their children’s language acquisition especially through repetition or simplification of their speech and through nonverbal communication (Snow, 1972). Whitehurst and colleagues initially described these strategies for use in language promotion and therapy contexts and summarized them into two central components, “PEER” and “CROWD” (e.g., Lonigan and Whitehurst, 1998). The acronym PEER (prompting, evaluating, expanding, repeating) describes the four key language support strategies. Prompting includes initiating language development strategies, i.e., primarily questions that are intended to stimulate the child to speak. The different types of prompting are summarized under the acronym CROWD (completion, recall, open-ended, wh-, and distancing questions). Reactive strategies (evaluating, expanding and repeating) are used to address, expand and repeat child utterances in order to provide the child with content feedback on different linguistic domains (Towson et al., 2020).

Many studies have found a positive effect of the use of LSS on children’s language outcomes. For example, the use of questioning strategies (open-ended, wh-, and distancing questions) has been shown to positively influence the verbal, cognitive and social skills of preschool children in general (Gunn and Hruska, 2017). The use of elicitations in small group settings, moreover, has been found to be supportive on children’s oral language acquisition (Hadley et al., 2022). However, recent studies have shown that the frequency of strategy usage in ECE institutions depends on how much the pedagogic situation is structured (Wildgruber et al., 2016; Beckerle et al., 2018; Burke Hadley et al., 2022) Further, it has been shown that caregivers and teachers underutilize LSS (Siraj-Blatchford et al., 2002; Beckerle et al., 2019). Thus, the integration of LSS into DR situations that are particularly structured is especially promising (Kammermeyer, 2019). DR is based on the following fundamental principles: “(i) evocative techniques that encourage the child to actively participate in reading and practice language, (ii) the use of informative feedback for the child regarding correct language use and (iii) progressive change where the adult adapts their reading style to the child’s developing linguistic abilities” (Pillinger & Vardy, 2022, p. 3).

Recent studies could not find a greater benefit from DR-interventions over other language support approaches, as the effect of DR was strongly dependent on the implementation fidelity (Ennemoser, 2017), i.e., on the actual extent to which professionals implemented LSS in DR interventions. However, in former literature, the effectiveness of DR-interventions is uncontroversial as several studies found positive effects of DR on children’s language outcomes (Pillinger and Vardy, 2022). For instance, there is evidence for positive effects of DR on young children’s narrative comprehension and nonword repetition skills (Holt and Asagbra, 2021). Neuman and Kaefer (2018) found positive effects on expressive, but not on receptive vocabulary using a pre-posttest control group design, although change in standardized scores remained insignificant. Effects are also strong with regard to bilingual children (Ennemoser et al., 2013) and children at risk for developmental language disorder (Holt and Asagbra, 2021). Furthermore, DR-interventions have the advantage of being strongly structured and efficient. DR is easy to implement and requires little preparation, is flexible in its implementation and adaptation of the linguistic content to the needs and interests of the children (Sigel and Inckemann, 2017) and is therefore of great benefit for professionalization programs.

### 1.3. Professionalization of caregivers in early childhood education

Among culturally and linguistically diverse children, previous ECE-based work has supported links between children’s L2 acquisition and caregivers’ language support. Moreover, the previous work has also challenged the findings by Busch and colleagues regarding the relevance of caregivers’ professionalization for the realization of language support in ECE. In their meta-analysis, Fitton et al. (2018) reported an overall positive effect of DR on bilingual children’s language outcomes, whether carried out by external experts or through training of the caregivers working in ECE. However, in contrast to additive language support interventions, integrated interventions implemented through professionalization of caregivers have the advantage of being highly frequent in everyday pedagogical situations (Kammermeyer, 2019). Thus, in general, effects of integrated language support could be reported, whereas effects of additive language support remained inconsistent (Egert and Hopf, 2016). Therefore, our goal was to implement LSS-based language support into ECD-groups through training of the caregivers.

In the recent years, many (inter-)national training programs for caregivers and teachers on the use of DR or LSS in general have been developed and evaluated (e.g., Neuman and Kaefer, 2018; Kammermeyer et al., 2019; Towson et al., 2020; Voltmer et al., 2021). Most studies investigated effects of the training programs only on children’s language acquisition for several language domains, mostly focused on expressive language skills. Thus, Blatter et al. (2020) conclude a positive effectiveness of different German training programs on expressive language outcomes of bilingual children and children in need of language support. In contrast to that, Voltmer et al. (2021) found effects of a caregivers’ training in LSS on monolingual and bilingual children’s morphological and syntactic language performance, but not on receptive and expressive vocabulary. So far, the investigation of trained caregivers’ competencies has been underrepresented in international studies evaluating training program effectiveness. The few studies available investigated the effectiveness of training programs on the usage of LSS using video analysis (e.g., Girolametto et al., 2003; Jungmann et al., 2013; Kammermeyer et al., 2019) and found positive effects on the language supportive interaction between children and caregivers.

Other studies from German-speaking countries set their focus on the standardized assessment of caregivers’ knowledge and skills rather than on observing the performance of language support (e.g., Roth et al., 2015; Beckerle et al., 2019; Lemmer et al., 2019), as different types of knowledge are considered prerequisites for, for example, the quality of teaching and students’ competencies (e.g., Yang et al., 2020). Models exist describing the competencies required for successful language support. For example, Hopp et al. (2010) conducted a “(psycho)linguistically oriented model” that aims to specify “competence criteria for language intervention based on psycholinguistic research” (p.609). Hopp et al. (2010) assume that caregivers have to be able to plan and reflect language support situations based on assessment or observation of linguistic skills of the children and theoretical knowledge about language acquisition in terms of the zone of proximal development (Vygotsky, 1978). Therefore, the model describes (1) theoretical *knowledge* about language acquisition, (2) *skills* needed to (3) *perform* language support. Accordingly, and in line with current research, both professional knowledge and skills of caregivers and teachers are considered prerequisites for the successful implementation of language support (Jungmann and Koch, 2017). Therefore, linguistically oriented models such as that developed by Hopp et al. (2010) provide a basis for planning specific training programs for language support. Training programs administered in studies investigating knowledge and skills focused on different content, i.e., on the usage of LSS (Beckerle et al., 2019) or on linguistic and practical knowledge (Roth et al., 2015; Lemmer et al., 2019). Overall, these studies found positive effects of intervention programs on the caregivers’ knowledge and theoretical competencies (Roth et al., 2015; Beckerle et al., 2019). For example, Roth et al. (2015) investigated caregivers’ linguistic and practical knowledge about language support and assessment before and after 12 days of training, which took place within a 10-month qualification phase. The authors found a significant increase of caregivers’ knowledge in both components, although lacking the comparison with a control group.

However, Fitton et al. (2018) criticize the lack of studies investigating the relation between effects on caregivers’ competencies and children’s outcomes and therefore an absence of evidence regarding competencies and knowledge needed to successfully implement language support. Only few studies investigated both caregivers’ and children’s competencies and found mostly positive effects on both areas (Buysse et al., 2010; Lemmer et al., 2019; Towson et al., 2020). In a recent study, Lemmer et al. (2019) assessed bilingual children’s expressive language outcomes and their caregivers’ language support competencies in a pre-posttest control-group-design before and after a caregiver training. The authors found improvements in children’s expressive sentence structure and caregivers’ knowledge about language support, but only when the interaction of time of measurement and group was considered. The distinction by group alone did not reveal significant differences. This finding indicates that caregivers and children in the experimental group improved their competencies more than participants in the control group.

However, Pillinger and Vardy (2022) and Fitton et al. (2018) point out that positive effects of training measures on children’s language outcomes often have to be interpreted cautiously, since most studies did not include control groups. In sum, most studies could demonstrate positive effects of caregivers’ training in LSS on children’s language acquisition and, if examined, on caregivers’ competencies. There are only a few studies regarding (a) the relation between effects on caregivers’ competencies and children’s outcomes, (b) effects on L2 acquisition of bilingual children with low LoE, and (c) studies including control groups. Therefore, these topics remain research gaps.

### 1.4. Research questions and hypotheses

The goal of the present study was to successfully implement language support into specialized ECD programs and thus, to strengthen language competencies and educational opportunities of participating preschool children without a regular childcare place. Therefore, we investigated whether bilingual children with low LoE to German as their L2 benefit from a caregivers’ training in LSS and DR. To follow this aim, we examined the following research questions:

1. Do the children’s receptive language scores (receptive vocabulary and grammar) differ from T_1_ (before caregivers’ training) to T_2_ (after implementation of language support)?
2. To what extent do the caregivers’ language support knowledge and skills change from T_1_ to T_2_ and do they influence the children’s receptive vocabulary and grammar skills?
3. Do the children’s receptive vocabulary skills differ from the ones of a control group of children who also visited ECD but without a caregivers’ training?

Considering recent findings, we expected (a) a significant improvement in receptive language skills of children (e.g., Jungmann and Koch, 2017; Blatter et al., 2020), with major increases in receptive grammar (Voltmer et al., 2021). Furthermore, we expected (b) caregivers’ language support knowledge and skills to increase after training and implementation of language support (Buysse et al., 2010; Lemmer et al., 2019; Towson et al., 2020). Understanding change on child-levels, we expected (c) caregivers’ competencies to influence language scores of the children. We also expected (d) children supported by trained caregivers to outerperform children of the control group (Lemmer et al., 2019).

## 2. Materials and methods

### 2.1. Participants and procedure

For the intervention group [IG], participants were recruited in 2022 from nine ECD groups in a western region of Germany. The initial sample consisted of 23 caregivers, who gave their consent of participating in the training program and the five-months intervention phase. The recruitment of participating children was conducted through recruiting caregivers. Seventy-six children were recruited for the IG, of whom 13 were excluded from the study because participants did not meet the inclusion criteria or did no longer attend the ECD group. In addition, data from 43 children from Busch et al. (2021) were used as a control group [CG]. Children in the CG attended ECD groups in 2017/18 with caregivers who did not complete a language support training. For both, IG and CG, inclusion criteria for participating children were the following: (1) children were in their last year before transitioning into school (2) children grew up bilingually^1^ with German as their L2, (3) they predominantly spoke another language than German at home; regular exposure to German began by entering the ECD program, and (4) they did not attend daycare before entering the ECD program. The final sample of participants of the IG who took part in the tests on at least one measurement occasion consisted of *n* = 54 children and *n* = 20 caregivers. For overall analysis, we selected all participants who participated in both measurement occasions in at least one of the measures focused in this study (*n* = 48 IG-children, *n* = 15 caregivers, *n* = 43 CG-children), as participants with missing data have been excluded for main analysis. For demographic information on the final analysis sample see Tables 1, 2.

**Table 1:** Sample characteristics of participating children.

Variable	Intervention group (*n* = 48)	Control group (*n* = 43)
Gender, *H*(%) female	20 (42%)	20 (47%)
Age (months), *M*(SD)	69.10 (4.75)	71.65 (10.96)
Heritage languages, *H*(%)		
Romanes	13 (27%)	–
Arabic	8 (17%)	–
Kurdish	5 (10%)	–
Spanish	4 (8%)	–
Turkish	4 (8%)	–
Somali	3 (6%)	–
Persian	2 (4%)	–
Other	9 (20%)	–
Region of origin^1^, *H*(%)		
Southeastern Europe	–	22 (51%)
North Africa	–	9 (21%)
Middle-East	–	8 (19%)
Subsaharan Africa	–	1 (2%)
Unknown	–	3 (7%)
LoE to German, *M*(SD)	5.79 (5.22)	7.22 (4.13)

^1^Since the data come from two different projects, heritage language was assessed for the intervention group, whereas heritage regions were collected for the control group. LoE, length of exposure. Age and LoE in months. H, absolute frequency. %, percent.

**Table 2:** Sample characteristics of participating caregivers.

Variable	*n* = 15
Gender, *H*(%) female	14(93%)
Age, *M*(SD)	39.8(13.66)
Educational background, *H*(%)	
Academic pedagogical	7(47%)
Non-academic pedagogical	5(33%)
Non-pedagogical	1(7%)
Teacher in-training	2(13%)
Work experience	8.47(6.86)

Age and work experience in years. H, absolute frequency. %, Percent.

**Figure 1:**
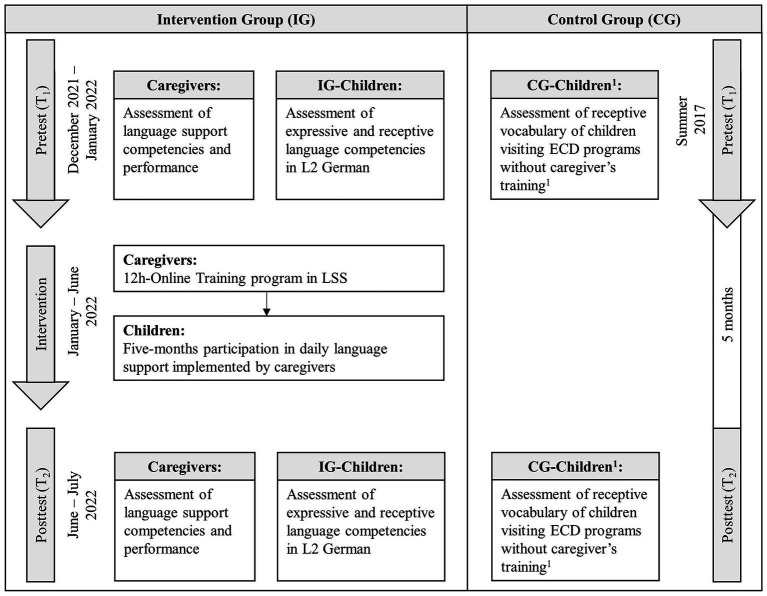
Study design. Language support was provided only during the children’s daily 3-h attendance of specialized ECD programs. Control group data were provided from Busch and colleagues (see Busch et al., 2021 for detailed information).

As CG and IG data were from two different projects, we inspected covariates descriptively and found no significant differences in the variables age and LoE using *t*-tests (age: *p* = 0.15, LoE: *p* = 0.15). The investigations on which the present study is based received a positive vote from the ethics committee of the TU Dortmund University. Children participated only after parents provided written informed consent. Study information and parental background questionnaires were translated into 15 heritage languages. To address our research goals, we chose a pre-post intervention study design. Figure 1 visualizes the process of the study. Assessments were conducted between January and July 2022 in two phases: initially at T_1_ and again 5 months later (T_2_). To additionally ascertain whether potential changes that occurred are a direct result of the intervention, we used CG data from a project investigating receptive vocabulary skills of children also visiting ECD programs but without a caregivers’ training (see Busch et al., 2021). We chose an inter-assessment interval of 5 months that was comparable to previous studies addressing the effectiveness of DR-based intervention (Pillinger and Vardy, 2022) and to the study by Busch et al. (2021) to compare our outcomes with a control group.

Eleven research assistants, all of whom (special) education students, were trained and supervised in child direct assessment procedures by the first and last author. At each measurement occasion, the respective child was tested in two sessions. Child direct assessments were administered with each child individually in separate rooms during ECD program hours and lasted around 30 to 45 min. Caregivers’ language support competencies were assessed before the training in LSS and after implementation of the language support.

### 2.2. Intervention

Caregivers were trained aiming at the use of LSS and performance of language support, particularly in DR, through a 12-h video-based training program. The training was delivered online on three separate days due to distancing polices during the COVID-19 pandemic. The training program consisted of three main modules: (1) milestones of language acquisition in bilingual children, (2) LSS and (3) DR. Following the classification of Beckerle et al. (2020) and the two central components of LSS, *PEER* and *CROWD* (e.g., Lonigan and Whitehurst, 1998), the training program mainly included five LSS in two global areas: reactive LSS and initializing LSS. Table 3 summarizes LSS used in the training program.

**Table 3:** Language support strategies following the classification of Lonigan and Whitehurst (1998) and Beckerle et al. (2020).

Language support strategy	Definition	Examples^1^
Reactive LSS		
Corrective feedback (evaluating/repeating/completion)	Indirect evaluation of an incorrect child’s utterance in different linguistic areas (e.g., phonologic, semantic, morphologic)	Child: “There is a mouse.” Adult: “Yes, there is a rabbit!” (Semantic Corrective Feedback)
Modeling (expanding)	Indirect modification or extension of a child’s utterance in different linguistic areas (e.g., phonologic, semantic, morphologic)	Child: “There is a rabbit.” Adult: “Yes, there is a rabbit. The rabbit has long ears.” (Semantic-syntactic Modeling)
Redirect (recall)	Returning a question of the child	Child: “What is that?” Adult: “Yes, what could that be?”
Initiating LSS		
Parallel talking	Accompanying actions and thoughts with language	“What color do I want to paint my house? Now I take the red crayon for the roof.”
Questions (prompting)	Open-ended questions, wh-questions, distancing questions	“Why do you think the boy is mad?”

^1^Own examples based on the definition of Lonigan and Whitehurst (1998) and Beckerle et al. (2020).

First, on two consecutive training days, the participants were mainly informed about language acquisition, LSS and basic principles of DR. Therefore, participants attended a two-hour lecture on milestones in bilingual language acquisition. The input contained information about bilingual language acquisition in general, and specifically in the different linguistic areas (phonetics, vocabulary, morphology and syntax, pragmatics and phonological awareness) as well as basic information about developmental language disorder. Small tasks were administered during the input phase, e.g., participants were asked to analyze the function of different types of verbs in exemplary sentences during the input on syntactic development.

The second module consisted of a three-hour block on (a) key situations conducive to language support and (b) information on the five main LSS (corrective feedback, modeling, redirecting, parallel talking and questions). For example, participants were asked to discuss everyday pedagogical situations suitable for language support in small groups. Afterwards, small input phases were given for each LSS. In addition, the participants were given short tasks to work on in small groups to find suitable LSS for exemplary situations. For example, the participants discussed appropriate feedback or modeling strategies to respond to exemplary non-target child utterances using a worksheet with examples of children’s expressions. Additionally, video material was provided to give examples of pedagogical situations suitable for language support (Baldaeus et al., 2021). The participants were asked to discuss the video examples and to evaluate the behavior of the caregivers and the LSS used in the videos. At the end of the second training day, an introduction to DR was given (1 h) using own video examples created to contrast good and poor practice for the use of LSS in DR situations. Afterwards, participants were asked to transfer their acquired knowledge into practice by implementing one DR-situation using LSS in their pedagogical work over the following days. After a three-day break for testing DR and LSS, the third day of training (4 h) was devoted to evaluate the first implementations into practice. Additionally, planning steps of DR and the adaptive use of LSS in DR were focused. Input was also given on phonological awareness and appropriate activities to promote the different language domains in everyday situations. Caregivers were instructed to regularly apply LSS in highly structured situations in their everyday pedagogical work. Additionally, during the five-month intervention phase, caregivers were regularly sent material packages with selected books and games suitable for language support.

### 2.3. Measures

Children’s L2-competencies were assessed using various standardized and informal diagnostic instruments for several language domains. Currently, there are no standardized language assessment tools available for children with L2 German and low LoE. Due to this lack of adequate measures for our population and following the suggestions by Rothman et al. (2022), we primarily used tasks that were normed for monolingual children interpreting raw scores rather than T-values for the present population. Additionally, we assessed caregivers’ language support competencies and performance. The assessment of language support competencies is described below, whereas the evaluation of language support performance using video analysis (following Beckerle et al., 2020) is still ongoing.

To assess children’s receptive and expressive vocabulary, we used two standardized German measurements [PDSS (Patholinguistic diagnostic in developmental language disorders; Kauschke and Siegmüller, 2009) and PPVT-4 (Peabody Picture Vocabulary Test; Lenhard et al., 2015)] and additionally a test for the assessment of specific vocabulary addressed during language support. To test children’s grammar skills, we used the TROG-D (German version of the Test for Reception of Grammar; Fox-Boyer, 2020) and spontaneous language samples by calculating specific grammar scores following the proceeding of Kauschke et al. (2022). To assess children’s narrative skills, we utilized the German version of the Multilingual Assessment Instrument for Narratives (MAIN; Gagarina et al., 2012). For assessment of phonological knowledge and awareness, we used the QU-NWR (LITMUS Quasiuniversal Nonword Repetition Tests; described in Grimm, 2022) and the German version of the Illinois Test of Psycholinguistic Abilities (P-ITPA; Esser and Wyschkon, 2010). In this study, we only report results for receptive vocabulary and receptive grammar skills. For the CG, only receptive vocabulary data is provided. Since we conducted tests which were normed for monolingual children, the comparison with the standardized *T*-values is ruled out. Raw scores were used as informative measures instead.

#### 2.3.1. Children’s receptive vocabulary

To assess children’s receptive vocabulary in their second language, we used the German adaption of the Peabody Picture Vocabulary Test (PPVT-4; Lenhard et al., 2015). The test contains 228 items, presented each with three distractors in ascending order of difficulty within a set of 12 items, respectively. For each item, the child is asked to select the picture that matches the word spoken by the research assistant. The session is terminated if the child makes eight or more errors in an item set. The test is standardized and normalized for children from 3;0 to 16;11 years. Overall internal consistency of the PPVT can be interpreted as excellent with *α* = 0.97.

#### 2.3.2. Children’s receptive grammar

To additionally assess children’s receptive grammar skills in their L2 in the IG, we used the German adaption of the Test for Reception of Grammar (TROG-D; Fox-Boyer, 2020). The test measures the understanding of 21 German grammatical structures, each tested in a block of four items, using different stimuli. For each item, the child is asked to point to the picture representing the sentence spoken by the experimenter. The session is terminated if the child makes one or more errors in five consecutive blocks. The TROG-D is standardized and normalized for children from 3;0 to 10;11 years. Overall internal consistency of the TROG-D can be interpreted as excellent with *α* = 0.90.

#### 2.3.3. Caregivers’ language support knowledge and skills

Assessment of theoretical and language support knowledge of the caregivers was conducted using the German SprachKoPF (Instrument for the standardized assessment of language support competence of pedagogical professionals; Thoma and Tracy, 2014). The SprachKoPF is an online questionnaire basing on the linguistic model for language support competence by Hopp et al. (2010). It assesses knowledge and skills of caregivers working in ECE. Linguistic knowledge (knowledge of different linguistic areas and language acquisition) and practical knowledge (knowledge of language assessment and support) are tested in 35 items using multiple choice and assignment tasks. Additionally, skills are tested using 18 tasks that describe concrete situations with case examples and videos. Participants can achieve an overall score between 0 and 1. Due to the guessing adjustment, individual negative values may appear. The test does not contain standard values. Internal consistency for the knowledge-component can be interpreted as good with *α* = 0.89, but is unsatisfactory for the skills-component with *α* = 0.64. Overall internal consistency for the total score can be interpreted as excellent with *α* = 0.9.

### 2.4. Data analysis

#### 2.4.1. Pre-analysis

Statistical analysis was performed using R (R Core Team, 2022, version 4.2.1). Through visual exploration of boxplots, we manually checked for outliers in the dependent variables (PPVT and TROG-D raw scores). No outliers were identified and the different scores were approximately normally distributed. Descriptive statistics were generated for all variables for both measurement occasions (*T*_1_ and *T*_2_). Raw scores were used as dependent variables. For caregivers, five different SprachKoPF-scores were calculated (total-score, knowledge-score, linguistic-knowledge-score, practical-knowledge score, skills-score).

*Hypotheses (a) and (b)*: To perform mean comparisons from *T*_1_ to *T*_2_, we first conducted paired t-tests for both language variables in the IG and for receptive vocabulary in the CG. For mean comparison of caregivers’ SprachKoPF-scores, we conducted the non-parametric Wilcoxon signed-rank test due to a small sample size (*N* = 15).

#### 2.4.2. Main analysis

For main analysis, we estimated separate multilevel linear mixed-effects models predicting fixed and random effects on children’s language scores (*T*_1_ and *T*_2_) using the lmerTest package (Kuznetsova et al., 2020). Children with incomplete observations were excluded from the main analysis. Alpha-error probability was set to 5%, i.e., we considered significance at α *<* 0.05. All metric variables were standardized using their grand mean and standard deviation. Children’s characteristics (age, gender, and length of exposure at *T*_1_) were used as covariates. For the SprachKoPF total-score, we calculated a mean score for each ECD group for *T*_1_ and *T*_2_ and assigned them to each participating child. For visualization of our results, and especially interpretation of cross-level interactions, we used estimated marginal means of fixed effects and created interaction plots using the emmeans-package (Lenth et al., 2022). To indicate the proportion of variance explained by random effects, intraclass correlation coefficients were calculated for all variables.

*Hypotheses (c)*: Addressing our first and second research questions about children’s receptive vocabulary and grammar growth in relation to caregivers’ language support competencies in interaction with time, we created two models, i.e., regressing on PPVT- and TROG-D-scores (repeated measurement, level 1 within-child). In the two models, we considered the effect of measurement occasion (time, level 1), caregivers’ SpachKoPF total-score (level 2: between children) nested in participants and caregivers, gender (level 2), age (level 2) and length of exposure (level 2) and a cross-level interaction between time and SprachKoPF total-score (level 2).

*Hypotheses (d)*: For our third research question about children’s receptive vocabulary growth compared to a control group, we regressed PPVT-scores on time, age, gender, length of exposure and group affiliation and the cross-level interaction with time (T_1_ and T_2_). For this model, we regressed children’s receptive vocabulary (repeated measurement, level 1) on measurement occasion (time, level 1), group affiliation (level 2), gender (level 2), age (level 2) and length of exposure (level 2) and a cross-level interaction between time and group affiliation (level 2).

## 3. Results

### 3.1. Overall changes in children’s language skills and caregivers’ language support competencies (hypotheses a and b)

Table 4 shows the intercorrelations between child characteristics and outcome variables at *T*_1_. Language variables correlate positively at a high level. Length of exposure did not correlate with any language variable, whereas the covariate *age* correlates with receptive vocabulary score at a low level, but not with receptive grammar score. There was a moderate negative correlation between receptive grammar score and caregivers’ SprachKoPF total-score.

**Table 4:** Intercorrelations between study variables at T1.

No.	Variable	1	2	3	4
1	Age	–	–	–	–
2	Length of exposure	0.04	–	–	–
3	Receptive grammar	0.10	0.22	–	–
4	Receptive vocabulary	0.32**	0.16	0.82***	–
5	Caregivers’ language support competencies	0.09	−0.26	−0.36*	−0.25

Significant correlations (***p ≤ 0.001, **p ≤ 0.01, *p ≤ 0.05) are depicted in bold.

Table 5 shows descriptive data of IG’s and CG’s language variables and of caregivers’ language support knowledge and skills. We found that children’s performance in all language variables in both groups increased over time, as well as caregivers’ language support knowledge and competencies in all scores. Paired-samples *t*-tests for mean comparison in the IG between T_1_ and T_2_ showed significant growth in both receptive grammar and receptive vocabulary, whereas change in receptive vocabulary scores in the CG was not significant. Regarding hypothesis (b) Wilcoxon’s Sign-Rank test of caregivers’ SprachKoPF scores from *T*_1_ to *T*_2_ revealed significant increases in all variables except linguistic knowledge.

**Table 5:** Descriptive statistics of dependent variables (raw scores) for children and caregivers at both measurement occasions (T_1_ and T_2_).

	Intervention group *(n = 48)*		Control group *(n = 44)*		Caregivers *(n = 15)*	
	*T*_1_	*T*_2_	*T*_1_	*T*_2_	*T*_1_	*T*_2_
	*M* (SD)	*M* (SD)	*M* (SD)	*M* (SD)	*M* (SD)	*M* (SD)
Receptive grammar Raw scores	16.85 (10.64)	24.67** (12.03)	**–**	**–**	**–**	**–**
Receptive vocabulary Raw scores	35.00 (22.29)	47.36* (23.3)	34.51 (23.12)	42.72 (22.07)	**–**	**–**
Language support competencies						
Total-score	–	–	–	–	0.27 (0.18)	0.34 (0.21)**
Knowledge	–	–	–	–	0.33 (0.2)	0.41 (0.25)*
Linguistic knowledge	–	–	–	–	0.42 (0.21)	0.42 (0.29)
Practical knowledge	–	–	–	–	0.28 (0.22)	0.41 (0.25)**
Skills	–	–	–	–	0.13 (0.18)	0.24 (0.17)*

Language support competencies were assessed using SprachKoPF (Thoma and Tracy, 2014). Paired-samples *t*-test was conducted for comparison of language outcomes at *T*_1_ and *T*_2_. Wilcoxon signed-rank test was conducted for comparison of language support competencies at *T*_1_ and *T*_2_. (**p ≤ 0.01, *p ≤ 0.05).

### 3.2. Effect of caregivers’ improvement in language support strategies on children’s language outcomes (hypothesis c)

Regarding hypothesis (c), we found time and caregivers’ improved scores from T_1_ to T_2_ in overall language support competencies to predict children’s receptive grammar in the IG. Also, caregivers’ language support competencies in general had a negative effect on children’s receptive grammar and receptive vocabulary scores. For other covariates on language variables in the IG, we found no influences. Analysis of change in receptive vocabulary showed no impact of caregivers’ language support competencies or the cross-level interactions with time. The statistical models are shown in Table 6.

**Table 6:** Changes in receptive grammar and vocabulary raw scores of participants in the intervention group.

Predictor	Model 1: receptive grammar (TROG-D)			Model 2: receptive vocabulary (PPVT)		
	*β*	SE	*p*	*β*	SE	*p*
(Intercept)	−0.45909	0.18714	0.018*	−0.24848	0.17660	0.165
Time (l.1)	0.88700	0.08473	<2e-16***	0.72767	0.08244	3.14e-16 ***
SprachKoPF (l.2)	−0.36196	0.10339	0.001***	−0.20388	0.09807	0.039*
Gender (f) (l.2)	−0.02964	0.27356	0.914	−0.20363	0.25445	0.428
Age (l.2)	0.11085	0.26405	0.677	0.04233	0.25097	0.867
LoE (l.2)	0.05509	0.13115	0.676	0.04774	0.12085	0.695
SprachKoPFxTime	0.19620	0.04154	3.74e-06 ***	−0.06278	0.04243	0.140
AgexGender (f)	0.16299	0.36330	0.656	0.26693	0.33814	0.434

*N* = 48. Significant fixed effects (***p ≤ 0.001, **p ≤ 0.01, *p ≤ 0.05) are depicted in bold. We used SprachKoPF total score in both models. l.1, level 1-variable. l.2, level 2-variable. ICCs of random effects for model 1: child: ICC = 0.77, Children nested in caregivers: ICC = 0.00. ICCs of random effects for model 2: individual: ICC = 0.83.

**Figure 2:**
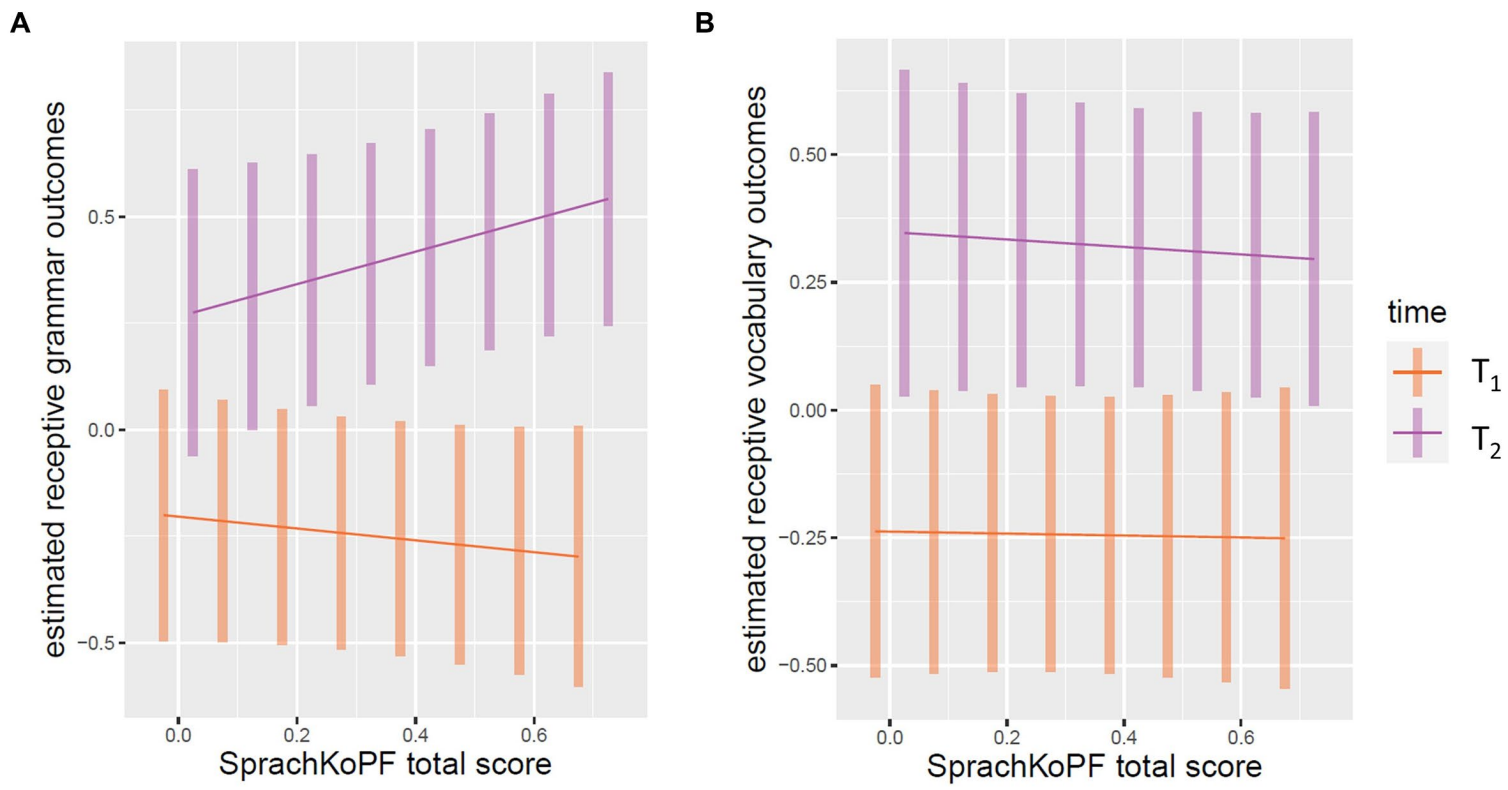
EMMIP plots for visualization of estimated marginal means for the influence of caregivers’ SprachKoPF total-score and time on children’s receptive grammar **(A)** and children’s receptive vocabulary **(B)** for the intervention group.

Visualizations of the estimated marginal means for Models 1 and 2 are shown in Figure 2. The left figure shows that children’s receptive grammar skills change as a function of an interaction between caregivers’ SprachKoPF scores and time, as there is a difference in the gradient of the two graphs. For the PPVT scores, we see no interaction between the caregivers’ SprachKoPF scores and time with respect to the children’s estimated receptive vocabulary scores.

### 3.3. Effect of group affiliation on improvement in children’s language skills (hypothesis d)

**Figure 3:**
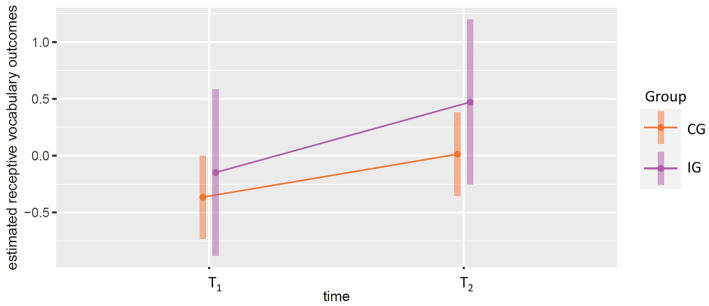
EMMIP plot for visualization of estimated marginal means for the influence of time and group affiliation on children’s receptive vocabulary for the intervention group (IG) and control group (CG).

To test hypothesis (d), we calculated a linear mixed-effects model using receptive vocabulary as the dependent variable and group and the interaction between group and time instead of caregivers’ language support competencies as covariates. Group membership was not linked to higher receptive vocabulary score, whereas time predicted higher scores. For more detailed results, see Table 7, visualization of estimated marginal means is shown in Figure 3. For all models, calculation of intraclass correlations indicated that, for all variables, the proportion of variance explained by intra-individual random effects is above 50 percent, whereas a negligible amount of variance could be explained by the assignment of child to caregivers (hence not considered in the multilevel structure of the statistical models).

**Table 7:** Changes in receptive vocabulary raw scores of participants in the intervention and control group.

Predictor	Model 3: receptive vocabulary (PPVT)		
	*β*	SE	*p*
(Intercept)	−0.24136	0.16871	0.161
Time (l.1)	0.34929	0.09562	0.000***
Group (IG) (l.2)	0.13399	0.20364	0.521
Gender (f) (l.2)	−0.16090	0.18506	0.387
Age (l.2)	0.19503	0.12741	0.130
LoE (l.2)	0.18349	0.09374	0.055
GroupxTime	0.20445	0.13480	0.133
AgexGender	0.20372	0.18240	0.267

*N* = 91. Significant fixed effects (***p ≤ 0.001) are depicted in bold. l.1, level 1-variable. l.2, level 2-variable. Participants of both groups were matched beforehand regarding length of exposure and age. ICCs of random effects: individual: ICC = 0.65. IG, intervention group. f, female.

Visualization of the estimated marginal means for Model 3 underlines the results shown in Table 7. There was no clearly identifiable interaction between group affiliation and time with respect to children’s estimated receptive vocabulary scores.

## 4. Discussion

The aim of the present study was to investigate the effectiveness of a training program for caregivers focusing on language support strategies and dialogic reading. This training focused on caregivers’ language support competencies in order to promote German language acquisition of children with little exposure to German as their second language. We assessed second language abilities of children and language support knowledge and skills of caregivers visiting specialized early childhood development programs in a pre-posttest intervention design. Our first findings suggest that caregivers’ language support competencies link to expanding bilingual children’s receptive grammar skills, but not to receptive vocabulary skills. Children’s receptive language and caregivers’ language support knowledge and skills increased over time, but caregivers’ competencies moderated gains in children’s receptive grammar only. Children’s receptive vocabulary skills could not be explained by caregivers’ gains in language support knowledge and skills. The comparison between intervention group and control group supported this finding, as there was no effect of group membership on children’s receptive vocabulary acquisition over time.

Overall, we found increasing receptive language scores in the intervention group from pre- to posttest in both areas. This is particularly encouraging since participating children visited the specialized early childhood development groups only for 3 h a day. As Rothman et al. (2022) suggested, we did not report standardized scores for all language measurements, due to the lack of comparability. Previous studies with bilingual children found standardized scores in pre-post analysis to remain flat, whereas raw scores changed significantly (e.g., Neumann et al., 2021). The described results regarding the increasing receptive language scores are consistent with most studies on the effectiveness of dialogic reading-interventions, as Pillinger and Vardy (2022) state in their review. Yet, most reported studies did not use control group design. To compare the results of our intervention group, we used control group data from Busch et al. (2021). The control group’s receptive vocabulary raw scores did not increase noticeably. Note, however, that a comparison of grammar scores with the control group was not possible in the present study, as Busch et al. (2021) did not investigate grammar. As expected, we found that the language variables at the first measurement occasion were strongly related. Length of exposure was not related to any of the language variables, while there was a small association between age and receptive vocabulary scores, but not with receptive grammar scores. This could be explained by the fact that the participating children in the intervention and control groups showed relatively little variation regarding age and length of exposure.

Regarding caregivers’ scores, we additionally found that practical language support knowledge and language support skills increased over time. This result is consistent with previous studies (e.g., Roth et al., 2015; Lemmer et al., 2019), which also found an increase of caregivers’ competencies who were trained in language support measured using the instrument SprachKoPF (Thoma and Tracy, 2014). Furthermore, inspecting caregivers’ language support competencies scores descriptively, we found greater variance of all scores at the posttest measurement occasion. This result is likely due to the fact that we tested caregivers’ language support competencies after a five-months implementation period (with *T*_2_ of the children) and not immediately after they participated in the training program (i.e., immediately after *T*_1_). Thus, we cannot make conclusions about short-term effects of the intervention. However, we do have information about long-term development of caregivers’ competencies, which provides insights into the quality of language support and the sustainable and lasting improvement of caregivers’ knowledge and skills.

In contrast to the training programs used by Roth et al. (2015) and Lemmer et al. (2019), our training was comparatively short with 12 h. However, linguistic knowledge did not increase from pre- to posttest. In their study, Roth et al. (2015) found that caregivers performed significantly better in both knowledge domains after 12 days of training, again finding the strongest effects related to practical knowledge. We explain this finding by the fact that practical content predominated in our training program. Due to time limitations, linguistic basic knowledge was only a subordinated topic, whereas practical knowledge about planning and evaluating language support situations dominated. A note on practical implication is in order here: As personnel shortage and time constraints prevail in ECE, it is hard to implement trainings for caregivers that are of longer duration. Even the implemented three online-training settings (of 4 h each) were partly hard to attend for the practitioners. All the more pleasant is the message that this short training block could already show significant effects on the children’s language acquisition.

Regarding the performance of language support, Hruska (2017) highlights the potential of video analysis for assessing the interaction between caregivers and children. In the present study, however, we do not report results on the usage of language support strategies. It could be the case that linguistic and practical knowledge and skills are not necessarily associated with performance of language support. A recent study of Kammermeyer et al. (2019), though, reports a significant increase in the usage of modeling strategies and complex questioning strategies after a training of caregivers in the usage of language support strategies.

Although improvements in both children’s language scores and caregivers’ knowledge and skills could be demonstrated in the present study, only change in receptive grammar could statistically be explained by improved caregivers’ outcomes, and thus we found no effect of caregivers’ knowledge and skills on increasing receptive vocabulary. Contrary to our expectations, there were general moderate negative effects of caregivers’ competencies on children’s language scores. As a possible reason for these somewhat puzzling results, it is conceivable that those caregivers working with children who have a particularly high need for language support had already taken a much more intensive interest in the topic of language support and therefore more experience before the intervention.

Only few other studies investigated both caregivers’ language support competencies and children’s language outcomes and found inconsistent, but mostly positive effects on both areas (Buysse et al., 2010; Lemmer et al., 2019; Towson et al., 2020). Whereas previous research could also find effects of general dialogic reading-interventions on bilingual children’s expressive vocabulary (e.g., Neuman and Kaefer, 2018), the present study once again supports previous findings about receptive vocabulary gains (e.g., Voltmer et al., 2021) by showing no effect of caregivers’ training in language support on receptive vocabulary outcomes of participating children. This result is supported by the fact that we found no substantial difference between intervention group and control group regarding the gain in receptive vocabulary. Due to the fact that we used the control group data from Busch et al. (2021), we were not able to make a group comparison for receptive grammar. However, with regard to receptive grammar outcomes, we can assume that the children’s language acquisition did actually benefit from the language support. One possible reason for the different effects of caregivers’ training in language support strategies found on vocabulary and on grammar acquisition may lie in the nature of acquisition on these distinct language domains itself and in the different kinds of presentations and repetitions needed for their intake. As we asked the caregivers to carefully manipulate the children’s input during intervention phase, it appears that the children’s intake of single words is not as tied to structured situations and structured input as it is to grammatical structures. These may be more dependent on language support strategies and structured situations like dialogic reading than vocabulary. In line with the argumentation of Voltmer et al. (2021), we assume that vocabulary acquisition “may depend less on lengthy supportive conversations” (p. 8) than grammar acquisition. Furthermore, the assessment of vocabulary is always item-based, since receptive or productive test procedures only test excerpts of the child’s vocabulary and, unlike grammatical phenomena, no general vocabulary performance is assessed.

Taken together, our preliminary findings indicate that preschool children with little exposure to German as their second language can benefit from a caregivers’ training program on language support strategies. As expected, we found greater increases in receptive grammar than in receptive vocabulary, and our study suggests a positive relationship between caregivers’ training in language support and children’s grammar acquisition.

### 4.1. Study limitations, future research, and practical implications

With regard to our methodological approaches, there are a number of challenges and limitations to our research which should be acknowledged. Since we aimed to implement language support into specialized preschool programs as frequently as possible, we chose to train caregivers to provide language support in everyday situations. Unlike additive language support programs, which are usually provided by external specialists, it is difficult to assess implementation fidelity for integrated interventions. Therefore, we instructed caregivers to use language support strategies and to engage in dialogic reading as often as possible (see similar procedures in Voltmer et al., 2021). To gain insight into the implementation of language support, we decided to ask the participating caregivers after the completion of the project how language support was and is still being provided after the end of the project. We assessed treatment checks afterwards with n = 13 caregivers. Most participants (69.23%) reported using language support strategies daily or several times a day after participating in the training. 30.77 percent of the respondents indicated that they consciously used language support strategies weekly or several times a week. In terms of performing dialogic reading, one participant reported performing dialogic reading several times a day. The remaining respondents reported performing dialogic reading once a week (61.53%) or several times a week (30.77%). 76.69 percent of respondents indicated that there were difficulties in conducting language support daily during the five-month project duration. Child-related factors such as low German language competencies or motivational issues were most frequently cited as difficulties as well as personnel shortage. With regard to long-term factors, we asked the professionals in the follow-up surveys about the frequency with which they now provide language support. The majority of professionals (84.62%) reported using language support strategies as frequently as they did during the project period, with two respondents reporting that they now use them more frequently. 38.46 percent of the respondents maintained the routine of dialogic reading as frequently as during the project period, four probands (30.77%) indicated that they did it less frequently after the completion of the project. Also four probands indicated they were now doing dialogic reading more frequently. Overall, respondents were satisfied with their daily language support practices and routines which have increased through participating in the research project and the training. 61.54 percent of the respondents stated that they were rather satisfied, 30.77 percent were even very satisfied. One participant was rather unsatisfied with the own language support practices.

Another limitation of our study concerns the control group design. Children of the control and of the intervention group who attended the specialized early child development programs both had in common, that they were recent immigrants who moved into socioeconomically disadvantaged neighborhoods and visited specialized preschool programs. Yet, the two groups were not fully comparable, as the control group data were collected between 2017 and 2018 and intervention group data were collected during the Covid 19 pandemic in 2022. Additionally, we had less detailed demographic variables for the control group than for the intervention group. Therefore, we were not able to include other variables than age, length of exposure to German and gender as control variables. As bilingualism is a diverse phenomenon with different conditions that have to be considered, Rothman et al. (2022) underline that “failing to have proper control reduces the meaningfulness of any found association.” (p. 2). Future studies may include at least the socioeconomic status as an important background variable as its influence on language is known (Hoff, 2003; Rowe, 2018). It should be noted, however, that the assessment of socioeconomic status is particularly difficult for children with a transnational family background due to a change in their living. It therefore can be assumed that most participating children in this study came from families classified with a low socioeconomic status in their current situation in Germany.

Other limitations concern the assessment of caregivers’ language support competencies: We only reported results referring to caregivers’ linguistic and practical knowledge and theoretical language support skills. We did not report caregivers’ usage of language support strategies in this study. Overall, we have referred to the linguistic model for language support competence of Hopp et al. (2010). They defined three central components of language support competence: *Knowledge*, *Skills* and *Performance*. We assessed knowledge and skills using a German online questionnaire (SprachKoPF; Thoma and Tracy, 2014). Caregivers’ performance of language support was also assessed in the presented project using videography of dialogic reading situations (following Beckerle et al., 2020). Qualitative and quantitative analyses of these caregivers’ language support performance are still ongoing. In the present study, we could not investigate the extent to which caregivers need linguistic knowledge to successfully conduct language support. Further research is needed to analyze the connection between linguistic knowledge and actual performance and to evaluate theoretical models about preconditions for successful language support. Another limitation relates to the reliability of the skills-score reported for language support competencies, which is unsatisfactory. Therefore, we reported the skills-score descriptively although for main analysis we used the total score of the SprachKoPF whose reliability scores can be interpreted as excellent. Additionally, the SprachKoPF was conducted online because of ongoing restrictions due to the Covid-19 pandemic. Therefore, we did not have external control about caregivers’ performance in the test.

Despite these limitations, our results suggest important practical implications. Overall, our training program was relatively short with 12 h, separated over 3 days. Additionally, we had a relatively high dropout of caregivers participating in our study. This underlines the difficulty of conducting training in preschool institutions, as caregivers were often not compensated for their participation by their employer. For this reason, we were also unable to offer substantial process support for the application of contents that were addressed in the training program. More intensive training is needed, which requires educational policy’s interest in further training of language support professionals and compensation for the caregivers by their employer. As a recent study on specialized preschool programs for children who did not attend daycare shows, the overall quality of language support was not rated as high (Busch et al., 2023). Bilingual children who receive little or no input in their second language (German) during preschool years are more often affected by educational disadvantage compared to their monolingual peers (Tienda and Haskins, 2011; Forrell and Bellenberg, 2022) and therefore need high-quality input in their second language before entering school.

In summary, our preliminary results support previous findings about the effectiveness of caregivers’ training in language support on bilingual children’s receptive grammar (Neuman and Kaefer, 2018; Lemmer et al., 2019; Voltmer et al., 2021), even for very short daily dosage of childcare. The findings contribute to a growing body of evidence, that language support strategies and the implementation of dialogic reading into pedagogical everyday situations is an effective way to support children’s language acquisition.

## Data availability statement

The raw data supporting the conclusions of this article will be made available by the authors, without undue reservation.

## Ethics statement

The studies involving human participants were reviewed and approved by ethics committee of the TU Dortmund University. Written informed consent to participate in this study was provided by the participants’ legal guardian/next of kin.

## Author contributions

JBo performed the statistical analyses and wrote the main part of the manuscript. A-LS and JBo collected the data of the experimental group. JBu and BL collected the data of the group that served as a control group for this study. A-LS administered the project and helped with writing few parts of the manuscript. JBu contributed the data, helped with analysis and wrote the chapter about specialized ECD programs. A-LS, BL, and JBu did proofreading. All authors contributed to the article and approved the submitted version.

## Funding

This study was part of the project “Basisfähigkeiten stärken – Qualifizierung, Diagnostik, Intervention,” [“Strengthening basic skills - qualification, diagnostics, intervention”] conducted from December 2021 to September 2022 and funded by the DKJS (Deutsche Kinder- und Jugendstiftung) as part of the funding program “AUF!leben – Zukunft ist jetzt.” The project was headed by A-LS. Control group data came from a project supported by the educational trust RuhrFutur (Mercator Foundation, 2018) through a grant to BL. We acknowledge financial support by Deutsche Forschungsgemeinschaft and TU Dortmund University within the funding program Open Access Costs.

## Conflict of interest

The authors declare that the research was conducted in the absence of any commercial or financial relationships that could be construed as a potential conflict of interest.

## Publisher’s note

All claims expressed in this article are solely those of the authors and do not necessarily represent those of their affiliated organizations, or those of the publisher, the editors and the reviewers. Any product that may be evaluated in this article, or claim that may be made by its manufacturer, is not guaranteed or endorsed by the publisher.

## Footnotes

^1^De Angelis (2007) suggests distinguishing between bilingualism and multilingualism for various reasons. Since the inclusion criterion of our study includes bilingual as well as multilingual children and we only include one first language that the children speak most at home, in the following we use the term “bilingualism” as a broader definition including bi- and multilingual children (Butler, 2013).
